# Glutamine Supplementation: The Pendulum Keeps on Swinging

**DOI:** 10.5005/jp-journals-10071-23216

**Published:** 2019-08

**Authors:** Subhash Todi

**Affiliations:** Department of Intensive Care and Internal Medicine, AMRI Hospital, Kolkata, West Bengal, India

## Abstract

**How to cite this article:** Todi S. Glutamine Supplementation: The Pendulum Keeps on Swinging. Indian J Crit Care Med 2019;23(8):350-351.

Immunonutrition has always been a hot topic of pro/con debate in critical care community. Even, international guidelines have changed their position from “for” to “against” immunonutrition specifically glutamine over years.^1,2^ As a clinician and researcher we need to introspect on the reasons for this controversy.

Firstly, glutamine level in the serum like many other metabolic, hormonal and vitamin molecules become deficient during stress (e.g vitamin C, vitamin D, selenium, vasopressin, cortisol) and severity of the deficieny of these molecules are consistently associated with poor outcome. Replacement of these deficient molecules, on the other hand, have not been shown to improve hard outcome markers like mortality in general intensive care population.^3^ This may be due to the fact that the association of deficiency with poor outcome, usually reflect an epiphenomenon and does not represent causality. In other words the deficiency is due to severity of illness rather than the other way round. It is also possible that glutamine and other similar molecules have low levels in critical illness as an adaptive response and trying to replace them may be maladpative and may not give desirable results.^4^

Secondly, a distinction need to be made between“ Nutritiopharmaceutical” which implies a pharmacological effect exerted by a larger pharmacological dose of the molecule as compared to physiological dose required for replacing the deficiency state. Thus the molecule, in this case glutamine, acts as a drug, where the desirable effect depends on pharmacokinetic/pharmacodynamic properties. This is evident in “REDOX” study which have shown no benefit and may be harmful effects of glutamine when higher doses were used, wheras earlier studies with lower doses have shown benefit.^5^

Thirdly, heterogeneity of nutrition trials make meaningful conclusion difficult, leading to variability in practice patterns. This heterogeneity results from (i) case mix of the trial population, as beneficial effects are found in trauma, burn, cancer surgery and pancreatitis patient and not in medically ill and septic patient treated with glutamine (ii) variable route of administration, enteral, parenteral or both. Enteral supplementation of glutamine was found to be beneficial in burn and trauma patient, wheras parenteral glutamine was found to be useful in post surgical and in patients on Total parenteral nutrition.^6^ Redox trial which did not show any benefit had both enteral and parenteral route of supplementation (iii) dose of glutamine. Lower doses of intravenous glutamine, e.g. 0.2–0.5 g/kg/day of glutamine dipeptivan have been found to be beneficial, whereas higher doses e.g. 0.5 g/kg/day was not beneficial (iv) interventional formula containing multiple nutritional products like glutamine, arginine, omega 3 fatty acid, antioxidant, etc. which makes it difficult to tease out benefecial effect of each ingredient (v) timing of adminstration, early vs late, with late supplementation in the disease course may not show beneficial effect (vi) variable outcome measures in different studies like infectious complications, length of stay, mortality. Most of the earlier non randomised studies have shown beneficial effects on decrease of infectious complications, predominantly pneumonia, decrease in insulin requirement and better glucose control and decrease length of stay, with very few studies showing beneficial effect on mortality. (vii) clinical severity and associated organ failure like septic shock, liver and renal failure, e.g REDOX study which included more severe patient showed a harmul effect. (viii) variable standardisation of calories and protein replacement targets in the control arm, and use of glutamine as a protein source or only as a drug.

Fourthly, contrary to the concept of personalised medicine, these interventions are applied irrespective of the baseline serum levels in patients e.g in REDOX study, glutamine levels were measured in a selected group of study population and only one third of them were found to be glutamine deficient, one third to have a normal level and one third having a high level of glutamine at baseline and adding further glutamine to these patients may have resulted in harmful effects. Intrestingly, baseline serum levels of glutamine are found to be low in patient population like burn, trauma, post surgery and pancreatitis, in whom glutamine supplementation have been shown to be beneficial. Compounding to this problem is the lack of ready availabilty of tests for serum levels of glutamine and other similar molecules. Morover serum levels may not reflect the tissue level of these molecules, and normalising serum levels may not be sufficient and we need tissue levels in the organ of interest. This phenomenon may reflect the discrepant results and lack of benefit of glutamine replacement in some studies.

Fifthly, more insight from bench and animal research elucidating the mechanism of action of these molecules are warranted and translational research to verify the findings of these basic research in human subjects need to be carried out before embarking on a large scale clinical trial with homogeneous case mix, early intervention with a specific formula at a specified physiological dose with a suitable control and having a meaningful clinical outcome.

The study published in this issue of the journal is an attempt at a translational research in this field where the researchers have done a commendable work in translating the concept of role of glutamine supplementation in decreasing bacterial translocation and improving gut permeability by measuring serum markers of gut permeability and endotoxin and endotoxin antibody. They have convincingly shown in a small clinical sample, the role of glutamine in maintaining gut integrity, though due to the small sample size they could not show any beneficial clinical impact of glutamine supplementation. Limitation of this study may be the presense of other confounding variable like alteration of gut microbiota, and underlying disease states those can also have effect on permeability, though randomisation should minimise these effects. Nonetheless, future clinical trials should take cues from these basic and translational research and if possible incorporate them in their research strategy. This may include measuring baseline serum/tissue levels of glutamine as inclusion criteria, measuring markers of gut permeability to monitor the beneficial effect of the replacement and measuring the turnover (synthesis and clearance) of the molecule to acheive a desired therapeutic serum/tissue level. The pendulum of glutamine will keep on swinging till we acheive these goals.

## References

[1] Singer P,, Blaser AR,, Berger MM,, Alhazzani W,, Calder PC,, Casaer MP (2019 Feb;). ESPEN guideline on clinical nutrition in the intensive care unit.. Clin Nutr..

[2] McClave SA,, Martindale RG,, Vanek VW,, McCarthy M,, Roberts P,, Taylor B (2009 May-Jun;). Guidelines for the Provision and Assessment of Nutrition Support Therapy in the Adult Critically Ill Patient: Society of Critical Care Medicine (SCCM) and American Society for Parenteral and Enteral Nutrition (A.S.P.E.N.).. JPEN J Parenter Enteral Nutr..

[3] Amrein K,, Schnedl C,, Holl A,, Riedl R,, Christopher KB,, Pachler C. (2014 Oct 15;). Effect of high-dose vitamin D3 on hospital length of stay in critically ill patients with vitamin D deficiency: the VITdAL-ICU randomized clinical trial.. JAMA..

[4] Van den Berghe G. (2013 Apr 18;). Low glutamine levels during critical illness–adaptive or maladaptive?. N Engl J Med..

[5] Heyland D,, Muscedere J,, Wischmeyer PE,, Cook D,, Jones G (2013 Apr 18;). A randomized trial of glutamine and antioxidants in critically ill patients. Canadian Critical Care Trials Group.. N Engl J Med..

[6] Novak F,, Heyland DK,, Avenell A,, Drover JW,, Su X. (2002;). Glutamine supplementation in serious illness: a systematic review of the evidence.. Crit Care Med.

